# Emergence and Magnitude of ML336 Resistance in Venezuelan Equine Encephalitis Virus Depend on the Microenvironment

**DOI:** 10.1128/JVI.00317-20

**Published:** 2020-10-27

**Authors:** Jasper Lee, Jyothi Parvathareddy, Dong Yang, Shruti Bansal, Kathryn O’Connell, Jennifer E. Golden, Colleen B. Jonsson

**Affiliations:** a Department of Microbiology, Immunology, and Biochemistry, University of Tennessee Health Science Center, Memphis, Tennessee, USA; b Regional Biocontainment Laboratory, University of Tennessee Health Science Center, Memphis, Tennessee, USA; c Laboratory Animal Care Unit, University of Tennessee Health Science Center, Memphis, Tennessee, USA; d Pharmaceutical Sciences Division, School of Pharmacy, University of Wisconsin—Madison, Madison, Wisconsin, USA; Cornell University

**Keywords:** ML336, Venezuelan encephalitis virus, *Alphavirus*, antiviral agents, next-generation sequencing

## Abstract

Venezuelan equine encephalitis virus (VEEV) is a New World *Alphavirus* that can cause neurological disease and death in humans and equines following transmission from infected mosquitoes. Despite the continued epidemic threat of VEEV, and its potential use as a bioterrorism agent, there are no FDA-approved antivirals or vaccines for treatment or prevention. Previously, we reported the discovery of a small molecule, ML336, with potent antiviral activity against VEEV. To further explore the population-level resistance profiles of ML336, we developed a whole-genome next-generation sequencing (NGS) approach to examine single nucleotide polymorphisms (SNPs) from virus passaged in dose escalation studies in a nonhuman primate kidney epithelial and a human astrocyte cell line, Vero 76 and SVGA, respectively. We passaged VEEV TC-83 in these two cell lines over seven concentrations of ML336, starting at 50 nM. NGS revealed several prominent mutations in the nonstructural protein (nsP) 3 and nsP4 genes that emerged consistently in these two distinct *in vitro* environments—notably, a mutation at Q210 in nsP4. Several of these mutations were stable following passaging in the absence of ML336 in Vero 76 cells. Network analyses showed that the trajectory of resistance differed between Vero and SVGA. Moreover, the penetration of SNPs was lower in SVGA. In conclusion, we show that the microenvironment influenced the SNP profile of VEEV TC-83. Understanding the dynamics of resistance in VEEV against newly developed antiviral compounds will guide the design of optimal drug candidates and dosing regimens for minimizing the emergence of resistant viruses.

**IMPORTANCE** RNA viruses, including Venezuelan equine encephalitis virus (VEEV), have high mutation rates that allow for rapid adaptation to selective pressures in their environment. Antiviral compounds exert one such pressure on virus populations during infections. Next-generation sequencing allows for examination of viruses at the population level, which enables tracking of low levels of single-nucleotide polymorphisms in the population over time. Therefore, the timing and extent of the emergence of resistance to antivirals can be tracked and assessed. We show here that in VEEV, the trajectory and penetration of antiviral resistance reflected the microenvironment in which the virus population replicates. In summary, we show the diversity of VEEV within a single population under antiviral pressure and two distinct cell types, and we show that population dynamics in these viruses can be examined to better understand how they evolve over time.

## Abstract (precis)

RNA viruses, including Venezuelan equine encephalitis virus (VEEV), have high mutation rates that allow for rapid adaptation to selective pressures in their environment. Antiviral compounds exert one such pressure on virus populations during infections. Next-generation sequencing allows for examination of viruses at the population level, which enables tracking of low levels of single-nucleotide polymorphisms in the population over time. Therefore, the timing and extent of the emergence of resistance to antivirals can be tracked and assessed. We show here that in VEEV, the trajectory and penetration of antiviral resistance reflected the microenvironment in which the virus population replicates. In summary, we show the diversity of VEEV within a single population under antiviral pressure and two distinct cell types, and we show that population dynamics in these viruses can be examined to better understand how they evolve over time.

## INTRODUCTION

Venezuelan equine encephalitis virus (VEEV) is an arbovirus belonging to the genus *Alphavirus* that causes a febrile illness in humans that may progress to life-threatening encephalitis but is often accompanied with symptoms including stupor, convulsions, and comas (1, 2). The largest and most recent major VEEV outbreak occurred in 1995 in northern Colombia and Venezuela and resulted in 85% of infected children showing neurological symptoms, compared to 15% of infected adults. In addition, the disease had a 5% case fatality rate (1). VEEV remains infectious when aerosolized and was developed as a biological weapon by the United States and USSR during the Cold War. Despite the ratification of the Biological Weapons Convention in 1972, which bans such activities, VEEV remains a potential biological threat with high priority (2, 3). Despite its potential threat to public health, there are currently no FDA-approved antivirals or vaccines for VEEV. Antivirals are of high importance, as epidemics of VEEV are unpredictable, and broad-ranging vaccine programs may not be feasible in all situations (4).

In recent years, we have worked on the discovery and subsequent development of promising chemical scaffolds which have potent antiviral activity against VEEV both *in vitro* and *in vivo*. In the first studies, we reported the antiviral activity of the hit compound, CID15997213, which showed a 50% effective concentration (EC_50_) of 0.8 μM against the VEEV strain TC-83 (5, 6). Later, we described compounds ML336 and BDGR-4, both of which showed increased potency in inhibiting TC-83 with respective EC_50_ values of 32 and 47 nM (6, 7). *In vitro* studies of CID15997213 and ML336 revealed mutations in VEEV TC-83 viruses when exposed to very high concentrations of each respective compound. These mutants were plaque purified and sequenced using the Sanger method. The identification of these mutations, Y102C and D116N in nonstructural protein 2 (nsP2) and the Q210K mutation in nsP4, along with our measurements demonstrating inhibition by ML336 in an *in vitro* assay using purified replication complexes, suggests the mechanism of action of these compounds interferes with the functions of those proteins in replication (8). These recent studies suggest that ML336 directly inhibits viral transcription; however, the precise mechanism is still under investigation (8). *In vitro* studies show that these mutations confer a 600-fold (Y102C) to a greater than 1,600-fold (Q210K) loss in the EC_50_ when tested with VEEV TC-83 and ML336 (6). In recent studies, we passaged VEEV TC-83 in the presence of BDGR-4, a derivative of ML336, and used next-generation sequencing (NGS) on samples at each successive passage to follow resistance at increasing concentrations of compound (7). Interestingly, in this limited NGS study, only the Q210K mutation emerged from the treatment of VEEV TC-83 with BDGR-4 in Vero 76 cells; however, a low frequency of a variety of other single nucleotide polymorphisms (SNPs) was discovered (7). Here, we explore the emergence and magnitude of resistance-conferring mutations to ML336 in two distinct microenvironments, nonhuman primate (NHP) kidney epithelial cells which lack IFN-α (Vero 76) and a human astrocyte cell line, SVGA (9).

The trajectory and dynamics of the emergence of antiviral resistance as a function of the compound concentration are an aspect of acute viral infections that has not been explored for many RNA viruses. This is due in part to the fact that there are only a handful of effective antivirals for treatment of acute RNA virus infections, such as influenza A viruses, poliovirus, and respiratory syncytial virus (10). RNA viruses, whether they cause acute or persistent infections, constantly generate random mutations in the genome during replication due to the high error rate of their polymerase and the lack of a proofreading function. On average, one error is made per every 10,000 nucleotides replicated (11). This leads to the creation of viral populations that contain many related, yet individually distinct, sequences within each of the virus particles (12). Whether these random mutations are positively or negatively selected during an infection clearly depends on the microenvironment in which a virus establishes an infection and replicates. Potential selective pressures include the host cell type (neuronal versus epithelial cell) and immune response (and type of immune cell present) as potential drivers of the evolution of these populations and fixing of specific advantageous mutations. The resistance to antivirals is also considered an evolutionary, adaptive process (13). Of note, the maintenance of resistance mutations in the virus population is often eliminated once drug treatment stops due to the tradeoffs inherent to the cost of resistance. However, this is not always the case, as observed with the seasonal H1N1 influenza virus for which a positive relationship was measured for fitness and resistance of the H274Y mutation with the neuraminidase inhibitor oseltamivir. Interestingly, serial passage of the N9 neuraminidase in a PR8 versus H1N1 A/WSN/33 (WSN) backbone with increasing oseltamivir concentration differed from that at passage 10 (1,954 μM), no mutants were observed in the N9 in the WSN backbone (14). This suggests cis-acting genetic factors may also influence the adaptive trajectory, which underscores the importance of assessment of each antiviral drug against the strains circulating. Lastly, sofosbuvir, a hepatitis C virus (HCV) NS5B polymerase inhibitor, was the first antiviral that included resistance data using NGS (15, 16). Establishment of robust NGS pipelines and examination of mutational variants, low or high, will be critical to interpretation of the relevance of variants over time in different cell types within an organ that may influence the metabolism and stability of the drug.

Here, we developed and deployed an NGS pipeline for whole-genome sequencing of VEEV TC-83 viral populations. In this study, we used this pipeline to define viral populations from mock-treated TC-83 and ML336-treated TC-83 over several escalating concentrations or passaging after removing ML336 entirely. The objective was to provide population-level assessments of information into the dynamics of resistance of ML336 over escalating doses. In agreement with reports of others, we show that virus populations change with respect to drug concentration, which is an important consideration in the design of effective antiviral treatment protocols (17, 18). In other words, in selection of the effective dose, one must consider not only the safety and elimination of the virus, but the treatment regimen that obviates emergence of resistance. Additionally, we examined the VEEV TC-83 resistance dynamics to ML336 in two distinct biological systems: a nonhuman primate epithelial cell line (Vero 76) and a human neuronal, astrocyte cell line (SVGA). We showed that the microenvironment influenced the timing of appearance, the constellation and the penetration of SNPs associated with ML336 resistance. We also showed that the nsp4 Q210 mutations are stable in the absence of compound over seven passages. As we design and test new small molecule derivatives for the alphaviruses, the pipeline developed and the results of these studies provide a framework and benchmark for the selection of compounds to advance.

## RESULTS

### Development of a whole-genome next-generation sequencing approach to assess single nucleotide variation in VEEV TC-83.

**FIG 1:**
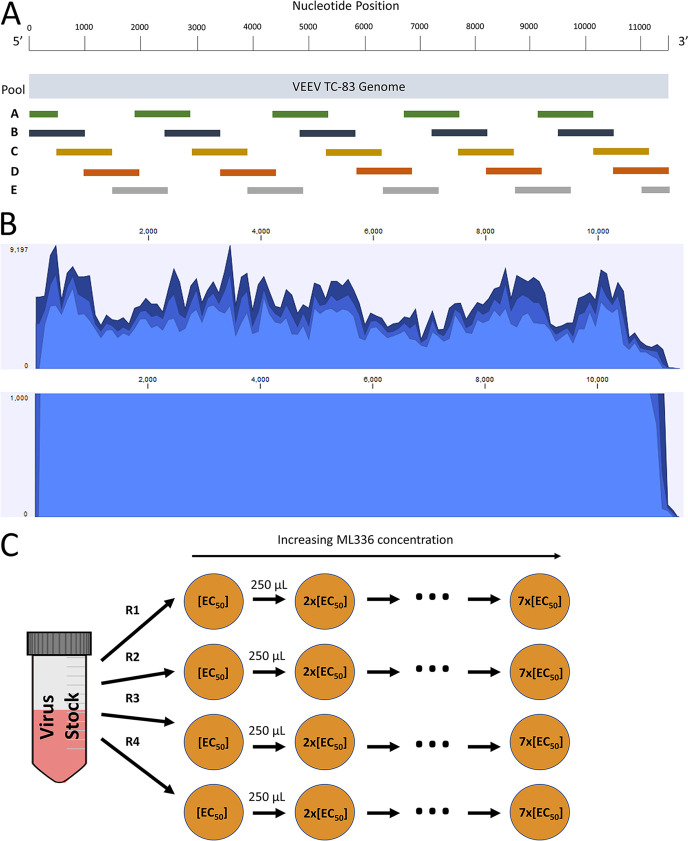
Methods for amplifying viral genomes for sequencing and detecting small nucleotide variations, and for passaging TC-83 through increasing concentrations of ML336. (A) Schematic for the tiling PCR method. Each colored bar represents an amplicon generated by a singular primer pair and is placed relative to the portion of the VEEV TC-83 genome it covers. Primer pairs of the same color were pooled together during PCR setup. Pools were combined prior to DNA purification. (B) Sample coverage graphs from applying the tiling method and deep sequencing the resulting amplicons. WT VEEV TC-83 was used to produce these graphs, using a total of 622,583 mapped reads. The top graph shows the depth and coverage throughout the genome, while the bottom graph shows coverage using a cutoff of 1,000 depth. (C) Schematic for the passaging of VEEV TC-83 in the presence of 2-fold increasing concentrations of ML336. The starting concentration was 50 nM. Vero 76 cells were grown for 2 days in a six-well plate and infected at the start of the experiment with 1 MOI. For each experiment, replicates are marked as R1, R2, R3, and R4.

In order to accurately identify single nucleotide polymorphisms in VEEV TC-83, we needed a method to generate enough sequences to provide sufficient coverage and depth across the entire genome to detect low-frequency variants. To achieve this goal, we developed a tiling method for whole-genome amplification in which we designed primers to create amplicons of approximately 1,000 or 500 bp long while ensuring the entire VEEV TC-83 genome was covered twice (Fig. 1A). In total, 25 primer pairs were identified as providing an efficient, and a similar level of, amplicon product using RNA isolated from our seed stock of wild-type (WT) VEEV TC-83 (Table 1). To ensure that the correct sequences were amplified, amplicons were purified by agarose gel electrophoresis and Sanger sequencing. This also provided a reference for the VEEV TC-83 genome based on Sanger sequencing. Primer sequences were optimized over time to produce equivalent amounts of product from the PCR. Initially, all amplicons were evaluated separately to ensure evenness in coverage. Second, we evaluated various approaches to pooling amplicons for sequencing. The optimal pooling noted from assessment of NGS data was one in which we sorted each primer pair into five different groups for PCR amplification (Fig. 1A). We pooled the PCR products from those groups together before purifying, preparing libraries, and conducting NGS.

**TABLE 1:** Primer pairs used to amplify the full genome of VEEV TC-83

Primer set no.	Direction^*a*^	Sequence
1	F	ATAGGCGGCGCATGAG
	R	CCCTTCGTAGCGACACG
2	F	ATAGGCGGCGCATGAG
	R	GCACAAGAATCCCTCGC
3	F	CAAGTCGCTGTTTACCAGG
	R	GGTAATGAGAGGTGACGGC
4	F	TATGGGAAGCCTTCAGG
	R	TACCTGTTTACGAACTCACG
5	F	CAGGAAAATGTTAGAGGAGC
	R	GAGCGCTCTGAGAGTACC
6	F	CCACCATTGTGTACAACG
	R	TTATCCATGGGTCGCC
7	F	GCTTTTGCTTGTCATGC
	R	CATAATTGCGCAGTGTACC
8	F	GTGGAAAACACTAGCCGG
	R	AACTTCCGTCTCTTCAAGTG
9	F	AGTCTATGACATGAACACTGG
	R	AAATGGTTCAATGATTGGG
10	F	GGTATGCAAACCGAAATCC
	R	TGTTCTGGACGTGAGGC
11	F	GAACAAAGATCGACTAACCC
	R	GAAACTCCATACTCTTTGCG
12	F	ATCCATGCCATGACTCC
	R	TTTCTTGGTCGAGGCG
13	F	CCGAGACTAACTCTTACTTCGC
	R	GCATTCCACATTAAAGGCC
14	F	TTGGAGATTTCGTATGCC
	R	GACGCGATGTCAGTTTCC
15	F	GAGAATTGCCCGTATTGG
	R	TGTCATCATCATGTTCATCG
16	F	TTCTGGAAACTGACATCGC
	R	GGTTTCTTAGCGGATGGC
17	F	CTGTTTAAGCTTGGCAAACC
	R	TCCCAGCACAATAGCG
18	F	GCTAACCTGACGTTCAAGC
	R	ATCCAGGCCATACTGCG
19	F	ACGACCCATTCTGGATAACC
	R	CCTTCTTTGTGCACTGGC
20	F	TGTTAGACTTCAGACTTCCTCG
	R	GGCGGCACAAATTGAC
21	F	GCAGTGCAGAGCATATCG
	R	GGCCTGTAAGTTGGAGTGC
22	F	ACCACAGATACCCTATGTCC
	R	CCAAGCTGTGGAAAGCGG
23	F	AGGAATGGATTCACCAGCC
	R	TGACAATCACCTTTGCACG
24	F	CTCCTGTGAATTTCAATGG
	R	AACAAAATCCGATTCGG
25	F	CCATCAGGGACTGCTACC
	R	AACAAAATCCGATTCGG

a F, forward; R, reverse.

We performed NGS on the pooled products to assess the coverage and depth of sequencing data we could obtain. We examined the data on low-frequency variants at a confidence level of 99.999%, according to standards outlined by Ladner et al. (19, 20). In the alignment step, we used a full genome sequence published on GenBank of VEEV TC-83 (accession number L01443.1, GI: 323714) to provide an initial reference (21). Thereafter, we used our consensus sequence as the reference. As shown in Table 2, there were two differences between the GenBank reference sequence and the consensus sequence of our seed stock virus. We noted nucleotide changes of T7208C and C11386T. These mutations are located in the nsP4 gene and the 3′ untranslated region of the genome, respectively, and as such, neither causes an amino acid change. The mapped reads showed 100% coverage throughout the genome (Fig. 1B). In addition, a more than 1,000× depth of coverage was obtained except at the very end of the 3′ untranslated region, surpassing the benchmarks necessary for confidence in identification of variants. In summary, the tiling method was sufficient in providing us the amount of sequencing reads necessary to detect and call variants.

**TABLE 2:** Summary of SNPs and amino acid changes between five samples of WT VEEV TC-83 and reference sequence (GenBank accession number L01443.1)^*a*^

Type	Nucleotide change	Amino acid change^*b*^	Gene	Sample 1 (%)^*c*^^,^^*d*^	Sample 2 (%)^*c*^^,^^*d*^	Sample 3 (%)^*c*^^,^^*d*^	Sample 4 (%)^*c*^^,^^*d*^	Sample 5 (%)^*c*^^,^^*d*^
SNP	C1151T	N369N	nsP1	7.26	5.11	5.14	5.92	6.34
Insertion	–2253A	NA	nsP2	1.86	3.83	2.42	–	–
Insertion	–2501T	NA	nsP2	3.17	3.12	3.44	–	–
SNP	C2627A	D326E	nsP2	–	1.51	1.08	–	–
Insertion	–2628A	NA	nsP2	4.61	6.36	5.49	5.01	2.78
SNP	T2873A	H408Q	nsP2	–	1.04	–	–	–
SNP	A5151C	I374L	nsP3	–	1.4	1.58	–	–
SNP	A5573G	S514S	nsP3	–	1.05	–	–	–
SNP	T7208C	P502P	nsP4	99.86	99.74	99.95	99.94	99.88
SNP	G7787A	G76R	C	1.36	–	–	–	–
Insertion	–9964T	NA	6K	–	2.98	3.24	–	–
SNP	C9987T	A52V	6K	–	1.79	1.7	–	–
SNP	T10759C	F253F	E1	1.82	–	–	–	–
Insertion	–10768T	NA	E1	–	–	–	1.52	–
SNP	G11149T	P383P	E1	1.57	1.05	–	–	–
SNP	C11386T	Noncoding	NA	99.75	100	100	100	100

a All SNPs appearing over 1% in at least one sample are shown.

b NA, not applicable.

c The percentage of SNPs present in that sample compared to the reference sequence.

d –, The SNP was not observed above 1% in that sample.

### Emergence and magnitude of VEEV TC-83 resistance to ML336 at increasing concentrations.

**FIG 2:**
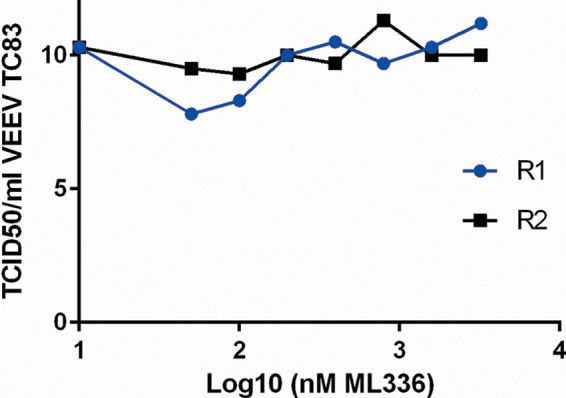
TCID_50_ data for R1 and R2 from passaging VEEV TC-83 in Vero 76 cells through increasing concentrations of ML336. For the biological replicates R1 and R2 (Fig. 2), we measured the titer of the virus after each passage at the time the supernatant was collected (48 hours postinfection [hpi]) and clarified. All supernatant was stored at –80°C until virus titers were assessed using the TCID_50_ assay, which is reported in this figure as TCID_50_/ml.

To determine how VEEV TC-83 evolves and adapts to ML336 at different concentrations, we infected VEEV TC-83 at a multiplicity of infection (MOI) of 1.0 in Vero 76 cells in a 6-well plate in which each well had 600,000 cells (Fig. 1C). This provided a sufficient population size to determine if any mutants were preexisting in the mock-treated group and for sampling the standing genetic variation/sequence space of the seed stock (approximately 10^9^/ml). VEEV-TC83 grows rapidly to very high titers, and hence, at this MOI, we were sampling approximately 0.6% of the available seed stock virions. All virus was removed following infection by washing with Dulbecco’s phosphate-buffered saline (DPBS), after which fresh medium or medium with compound was added. We started the first passage at 50 nM, a concentration slightly above the EC_50_ (30 nM) and increased the concentration of ML336 2-fold at each passage, namely, 100-, 200-, 400-, 800-, 1,600-, and 3,200-nM concentrations (6). The experiment included four biological replicates (R1 to R4). As a control, VEEV TC-83 was passaged in the absence of ML336 in each experiment in an identical manner as the virus with compound. RNA was isolated from twice-clarified supernatant at each passage, and we sequenced each sample as described in Materials and Methods. We also assessed the amount of virus after each of the passages R1 and R2 (Fig. 2).

**FIG 3:**
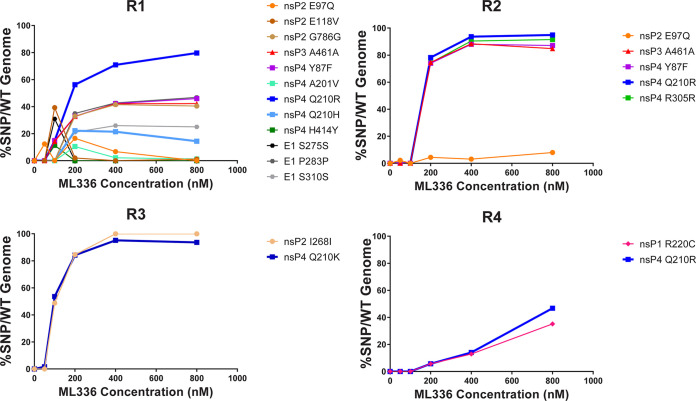
Four biological replicates of VEEV TC-83 passaged in Vero 76 cells over escalating doses of ML336 reveal four distinct paths to a common solution of resistance. Each well with approximately 600,000 cells was infected with an initial MOI of 1, washed to remove any unbound virus, and then passaged in the presence of 50-, 100-, 200-, 400-, 800-, 1,600-, or 3,200-nM concentrations. The first five passages (50, 100, 200, 400, 800) of each replicate, R1, R2, R3, and R4, are plotted. Each SNP that was found in at least 10% of all reads in at least one passage was defined and graphed for each passage/ML336 concentration. Each unique SNP is represented by an individual color.

Each replicate at each concentration was evaluated for SNPs (Fig. 3, Table 3). We only considered SNPs that reached a percentage of over 10% of the population, as those that emerged between 1 and 10% were usually not stable and did not penetrate over time. Replicate 1 (R1) produced the highest number of SNPs, with 12 in total. In R2, four SNPs became dominant in the population by the third passage. Replicates R3 and R4 only saw two major SNPs. The identities of the SNPs detected were distinct; however, R1, R2, and R4 all showed a Q210R mutation in nsP4. R1 and R2 shared an E97Q SNP in nsP2 and a Y87F SNP in nsP4, as well as a synonymous mutation at position A461 in nsP3. R1 was the only replicate where a significant percentage of SNPs in structural genes emerged, all within in the E1 gene and all synonymous mutations. A second nsP4 Q210 mutation, with an amino acid change into histidine, was observed in R1 at a lower frequency than Q210R. The emergence of E118V and G786G in nsP2, and A201V and H414Y in nsP4 were also unique to R1. A nucleotide change at R305 in nsP4 was found in R2 only. The I268I mutation only appeared in R3, while the only nsP1 mutation, R220C, was found in nsP4. No SNPs above our variant calling cutoff were detected in virus-only controls where TC-83 was passaged without the presence of ML336 (data not shown).

**TABLE 3:** Summary of the percent of SNPs and amino acid changes found at increasing concentrations of ML336 in Vero 76^*a*^

Nucleotide change	Amino acid change	Gene	Replicate	50 nM	100 nM	200 nM	400 nM	800 nM	1,600 nM	3,200 nM
C692T	R220C	nsP1	R4	–	–	5.65	12.95	35.18	50.61	33.17
C920T	I292I	nsP1	R3	–	9.59	3.59	–	–	–	–
G1938C	E97Q	nsP2	R1	12.42	–	16.55	6.72	–	20.34	–
			R2	2.25	–	4.42	3.08	8.01	15.57	20.6
A2002T	E118V	nsP2	R1	–	39.24	2.06	–	1.22	1.63	2.16
T2453C	I268I	nsP2	R3	–	48.76	84.54	>99.99	>99.99	>99.99	>99.99
T4007C	G786G	nsP2	R1	–	–	32.99	41.52	40.43	39.84	31.60
G5414A	A461A	nsP3	R1	–	14.24	32.72	42.12	42.28	43.81	38.16
			R2	–	–	74.60	88.35	84.82	82.66	66.62
A5962T	Y87F	nsP4	R1	–	14.9	32.93	42.26	45.95	48.88	51.23
			R2	–	–	73.84	88.01	87.14	88.04	84.43
C6330A	Q210K	nsP4	R3	1.67	53.61	84.16	95.15	93.62	95.72	95.59
A6331G	Q210R	nsP4	R1	–	14.9	56.25	70.89	79.65	85.50	89.94
			R2	–	–	78.22	93.61	94.89	96.37	95.06
			R4	–	–	5.72	14.04	46.75	85.10	85.12
A6332T	Q210H	nsP4	R1	–	–	22.20	21.48	14.46	9.73	5.14
G6617A	R305R	nsP4	R2	–	–	74.32	90.42	91.54	92.68	92.59
C6942T	H414Y	nsP4	R1	–	11.4	–	–	–	–	–
C7367T	R555R	nsP4	R1	1.03	–	10.00	2.15	–	–	–
A10825T	S275S	E1	R1	–	30.90	–	–	–	–	–
C10849T	P283P	E1	R1	–	–	34.99	42.79	46.83	48.27	45.84
C10930T	S310S	E1	R1	–	–	21.08	25.95	25.00	25.91	24.81

a Dashes represent data below the limit of detection.

In R1, R2, and R4, SNPs appeared at a concentration of 100 nM or 200 nM ML336 and increased rapidly up to 400 nM, after which the magnitude of SNP penetration appeared to plateau, especially if the percentage of the SNP in the population reached over 90%. However, in R1, a few SNPs, including H414Y (nsP4) and S275S (E1), appeared at 100 nM and then immediately disappeared from the population in subsequent passages. The E97Q mutation in R1 and R2 showed two distinct trajectories, where it would either disappear and reappear or slowly increment in the population. Both SNPs that reached consensus in R3 appeared at significantly high percentages at 100 nM. Lastly, both major SNPs found in nsP4 exhibited trajectories where both SNPs increased in percentage at a much slower rate, with the Q210R mutation only peaking at 3,200 nM (Table 3). In summary, these results show that TC-83 evolved resistance to ML336 through a variety of paths but converged in a resistance solution at the Q210 amino acid position.

### Mutant populations stayed consistent when passaging in the absence of ML336.

**FIG 4:**
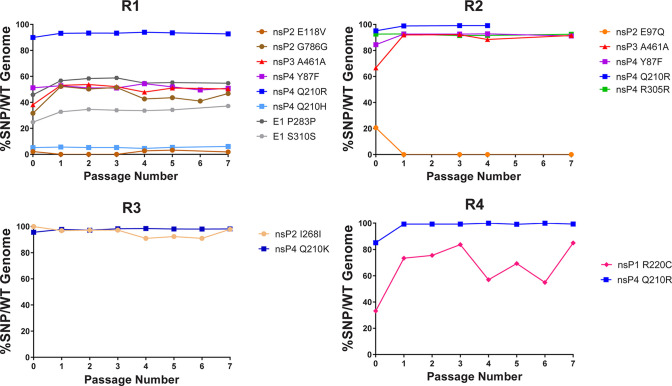
Most SNPs show stability over seven passages in the absence of ML336. Supernatant from VEEV TC-83/3200 was used to infect a six-well plate seeded with Vero 76 cells for 1 h at an initial MOI of 1. After 1 h, medium was removed and replaced with fresh complete MEM. Supernatant from each passage was collected at 24 h and subjected to NGS as described in Materials and Methods. Then, 250 μl of supernatant was used to infect new cells (passage 2). Passages were repeated 5 additional times. The *y* axis shows the percentage of SNPs relative to the WT population.

To determine whether the mutations generated in the previous experiment were stable, each replicate was passaged an additional seven times in Vero 76 cells without the presence of ML336. The starting virus sample used was from the 3,200-nM supernatant from each replicate in the previous experiment (referred to as VEEV TC-83/3200), which was inoculated at an MOI of 1. At each passage, virus supernatant was collected and sequenced in the same manner as before. As shown in Fig. 4 and Table 4, the major mutations in each population remained similar through each passage, although there were two interesting cases. First, the nsP2 E97Q mutation in R2 disappeared from the population after one passage and did not reappear. Second, the R220C amino acid change in nsP1 in R4 fluctuated in percentage through the seven passages, but we observed that all the percentages were higher than when the virus was passaged in the presence of ML336. In conclusion, these combinations of mutations were stable and did not cause a loss in fitness to the viral population in the absence of ML336, since they were maintained well through repeated passages.

**TABLE 4:** Summary of the percent of SNPs and amino acid changes for VEEV TC-83/3200 passaged in the absence of ML336^*a*^

Replicate	Nucleotide change	Amino acid change	Gene	3,200 nM (original)^*b*^	P1	P2	P3	P4	P5	P6	P7
R1	A2002T	E118V	nsP2	2.16	–	–	–	2.69	3.21		1.83
	T4007C	G786G	nsP2	31.60	52.29	50.26	51.48	42.64	43.57	41.04	46.78
	G5414A	A461A	nsP3	38.16	53.1	53.7	52.11	47.9	51.01		50.37
	A5962T	Y87F	nsP4	51.23	52.8	51.19	51.04	54.33	51.9	49.53	50.85
	A6331G	Q210R	nsP4	89.94	93.13	93.3	93.24	93.93	93.53		92.69
	A6332T	Q210H	nsP4	5.14	5.62	5.25	5.23	4.57	5.35		6.07
	C10849T	P283P	E1	45.84	56.7	58.35	58.8	54.84	55.24		54.76
	C10930T	S310S	E1	24.81	32.72	34.64	33.95	33.61	34.25		37.17
R2	G1938C	E97Q	nsP2	20.6	–		–	–			
	G5414A	A461A	nsP3	66.62	91.92	92.47	92.13	88.45			91.48
	A5962T	Y87F	nsP4	84.43	92.56		92.59	92.76			91.96
	A6331G	Q210R	nsP4	95.06	98.85		99.1	99.07			
	G6617A	R305R	nsP4	92.59	92.71		91.46	91.55			92.46
R3	T2453C	I268I	nsP2	–	96.81	97.14	97.22	90.92	92.32	90.92	97.84
	C6330A	Q210K	nsP4	95.59	97.8	97.13	98.25	98.45	98.12	98.02	98.16
R4	C692T	R220C	nsP1	33.17	73.31	75.47	83.65	56.93	69.25	54.86	84.95
	A6331G	Q210R	nsP4	85.12	99.25	99.30	99.26	99.99	99.13	99.99	99.37

a P1 to P7 represent each successive passage. Blank cells indicate that the depth of coverage was too low to analyze at that nucleotide position. Dashes represent data below the limit of detection.

b Values under the 3,200 nM column are the same as those in the 3,200 nM column in Table 3.

### Passaging TC-83 through increasing concentrations of ML336 in the SVGA cell line.

The Vero 76 cell line is deficient in type-I IFN production, thus creating a cellular environment that allows viruses to easily proliferate (9). In order to test the emergence of resistance to ML336 in an environment that more closely resembles that of a natural infection and creates a higher barrier to replication, we selected the SVGA cell line as our model. The SVGA cell line is derived from human astrocyte cells, and VEEV has been shown to replicate in astroglia in the central nervous system (22, 23). To show SVGA was a viable model for VEEV TC-83 infection, we performed a time course infection of SVGA cells. TC-83 reached a titer of over 1 × 10^9^ median tissue culture infectious dose (TCID_50_)/ml by 24 h, which is in line with titers from infection of Vero 76 cells (data not shown). Thus, we showed that TC-83 was able to productively infect the cell line.

**FIG 5:**
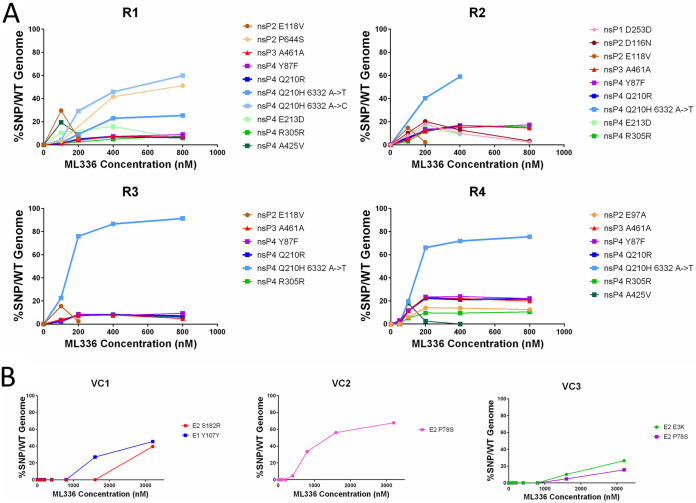
Passaging TC-83 in SVGA cells through increasing concentrations of ML336 showed that ML336 exerted a stronger environmental pressure. Each well was infected with an initial MOI of 0.1. All mutations appearing in at least 10% of all sequence reads found in at least one passage were included, plotting their percentage in the population over each ML336 concentration. (A) The first five passages of each experimental replicate were plotted. A distinction has been made between the two Q210H mutations to show that the A to T mutation is more common. (B) Graphs of three virus-only controls, showing all passages. All mutations occurred in genes coding for envelope proteins.

With this result, we passaged and sequenced TC-83 in SVGA in the same manner as with Vero 76, using the same ML336 concentrations as before. To serve as a control, we passaged the virus without the addition of ML336. We sequenced all four replicates at each concentration (Fig. 5A, Table 5) and three replicates of virus without ML336 (Fig. 5B, Table 5). In all four replicates passaged with ML336, the Q210H mutation arose to become the most dominant mutation, again appearing within 100 to 200 nM of ML336. In addition, the Q210R mutation also appeared in all replicates at a similar timing, though in each replicate it followed a trajectory where it was less prominent than the Q210H mutation. Interestingly, in R1, we discovered a second nucleotide mutation in the Q210H mutation in which cytosine instead of the usual thymine arose at nucleotide position 6332, and the cytosine mutant became more dominant than the thymine as ML336 concentration increased in subsequent passages. The trajectories of each replicate compared to each other were slightly more uniform, as the A461 synonymous mutation in nsP3 and the Y87F and R305 mutations in nsP4 appeared in all replicates, starting at 100 nM. The A425V mutation in nsP4 was shared between R1 and R4, the E213D mutation in nsP4 was shared between R1 and R2, and the E118V mutation was shared between all replicates except R2. SNPs unique to each replicate mostly occurred in nsP2, where the P644S (R1), D116N (R2), and E87A (R4) mutations appeared. R2 also contained a D253D mutation in nsP1, and mutations in E1 appeared in R1.

**TABLE 5:** Summary of percent of SNPs and amino acid changes found at increasing concentrations of ML336 in SVGA^*a*^

Nucleotide change	Amino acid change	Gene	Replicate	50 nM	100 nM	200 nM	400 nM	800 nM	1,600 nM	3,200 nM
C803T	D253D	nsP1	R2	0	8.16	17.91	9.82	2.33	–	–
A1939C	E97A	nsP2	R4	0	6.02	14.06	13.86		14.62	18.18
G1995A	D116N	nsP2	R2	0	10.34	20.31	12.81	3.28	–	–
A2002T	E118V	nsP2	R1	0	29.64	5.62	–	–	–	–
			R2	0	14.83	2.34	–	–	–	–
			R3	0	15.53	2.46	–	–	–	–
C3579T	P644S	nsP2	P1	0	3.19	–	41.57	51.17	55.36	45.54
G5414A	A461A	nsP3	R1	0	1.56	4.02	7.48	6.6	10.36	11.32
			R2	0	4.9	12.04	16.63	14.59	22.94	33.26
			R3	0	4.09	7.17	7.91	4.82	7.53	12.01
			R4	0	11.74	23.06	22.09		21.6	35.88
A5962T	Y87F	nsP4	R1	0	1.27	3.95	7.28	8.94	7.55	9.99
			R2	0	4.15	13.55	14.82	17.75	25.65	34.43
			R3	0	3.21	8.42	7.52	9.32	9.72	13.65
			R4	3.19	11.29	23.38	23.81		25.35	34.12
A6331G	Q210R	nsP4	R1	0	1.19	4.84	7.15	6.45	8.01	10.61
			R2	0	–	12.28	16.56	–	19.12	36.27
			R3	0	2.44	8.17	8.11	6.99	9.34	13.56
			R4	0	11.51	22.38	21.26		24.85	30.37
A6332T	Q210H	nsP4	R1	0	1.71	9.18	22.94	25.32	35.36	35.74
			R2	0	–	40.32	58.91	–	77.3	61.54
			R3	0	22.55	75.97	86.54	91.33	89.24	84.69
			R4	0	19.6	66.07	71.83		71.46	66.67
A6332C	Q210H	nsP4	R1	0	4.38	29.28	45.75	59.85	55.26	51.95
A6341T	E213D	nsP4	R1	0	10.52	–	15.88	5.34	–	–
			R2	0	–	15.08	9.31	–	–	–
G6617A	R305R	nsP4	R1	0	–	–	4.95	7.69	6.87	12.16
			R2	0	3.03	11.55	16.77	15.51	21.85	32.26
			R3	0	3.27	7.79	8.04	5.82	9.31	12.64
			R4	0	5.24	9.63	9.49		11.18	13.26
C6976T	A425V	nsP4	R1	0	19.45	9.03	–	–	–	–
			R4	1.81	17.93	2.47	–		–	–
G8570A	E3K	E2	R1	0	–	–	–	–	4.5	13.11
			VC3	0	–	–	–	–	10.09	26.4
C8795T	P87S	E2	VC2	0	–	–	4.63	33.49	56.07	67.62
			VC3	0	–	–	–	–	4.81	15.54
A9107C	S182R	E2	VC1	0	–	–	–	–	–	39.52
A9210G	Q216R	E2	R1	0	–	–	–	–	5.31	11.83
C10321T	Y107Y	E1	VC1	0	–	–	–	–	26.96	45.45

a Dashes represent data below the limit of detection.

Lastly, in the virus-only controls, the only SNPs that emerged were located in genes coding for structural proteins (Fig. 5B, Table 5). Notably, the E3K mutation in the E2 gene was found in both R1 and virus control 3 (VC3), while the P78S mutation was found in both VC2 and VC3. A unique Y107Y mutation also emerged in VC1. Taken together, these results show that the mutation trajectory of TC-83 when subjected to ML336 diverges in SVGA cells compared to Vero 76 cells.

### NGS of VEEV TC-83 in brains from a lethal mouse model treated with ML336 did not detect resistance variants.

**FIG 6:**
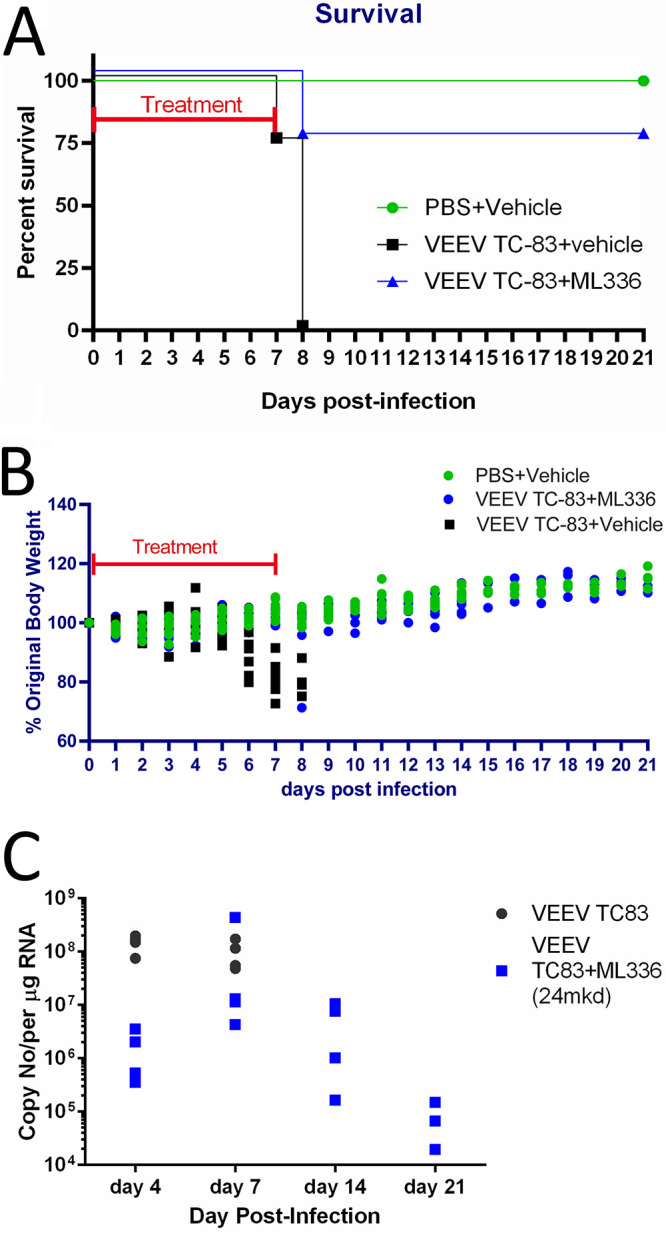
Time points chosen for NGS. *In vivo* efficacy of ML336 tested in C3H/HeN mice challenged with VEEV TC-83. Mice were i.n. infected with TC-83 and treated with either ML336 (i.p.; 24 mg/kg/day) or vehicle for 8 days starting from 0 dpi. (A and B) Survival (A) and weight change (B) were monitored over 14 days. The survival rates were compared between the ML336-treated and placebo-treated groups using Log-rank (Mantel-Cox) test. (C) RT-qPCR data for each individual mouse. *** represents *P* < 0.001. Mouse brains were collected on days 4, 7, 8, and 14 for RNA extraction.

Next, we aimed to examine the potential of ML336 resistance *in vivo*, using the mouse strain C3H/HeN, in which TC-83 can cause a lethal infection at 1 × 10^7^ PFU (7, 24). Previously, we reported that intraperitoneal (i.p.) administration of ML336 at 25 mg/kg/day, twice per day (BID), gave complete prophylactic protection (*n* = 8) when challenged with VEEV TC83 (24). Based on these data, we selected time points at days 4, 7, 8, and 14 to examine brains for the presence of viral RNA by MiSeq NGS using the aforementioned approach developed for our *in vitro* studies. Groups of C3H/HeN mice were infected with 1 × 10^7^ PFU TC-83 and treated with either 24 mg/kg/day ML336 or vehicle, starting 2 h before infection for 8 days. Mice were monitored for survival and weight change (Fig. 6A and B).

As shown previously, treatment of mice with ML336 at 24 mg/kg/day over 8 days provided protection (*n* = 7/8) through day 14, with only a single mouse succumbing to disease at 8 dpi (Fig. 6A) (6, 24). In the group with VEEV TC83, a sharp decline in body weight was observed beginning at 5 dpi, whereas the ML336-treated mice continued to have a similar level of weight gain as the noninfected mice (Fig. 6B). Brains from sacrificed mice at 4, 7, 8, or 14 dpi were homogenized, and RNA was extracted and processed for quantification through reverse transcriptase quantitative PCR (RT-qPCR) to confirm the amount of virus present before NGS (Fig. 6C). SNPs detected in the brains of TC-83-infected mice at percentages above 10% are shown in Table 6. The ML336-treated mouse brains did not result in a sufficient number of sequencing reads that allowed for analysis and featured uneven depth of coverage; however, we were able to pull SNPs from the 6K portion of the genome for one sample where SNPs were observed above 10% penetration. None of the SNPs found in this experiment had been previously found to confer resistance to ML336.

**TABLE 6:** Summary of point mutations and amino acid changes found in TC-83 extracted from C3H/HeN mouse brains

Nucleotide change	Amino acid change^*a*^	Gene	Treatment	Sample	Day euthanized	SNP percentage
T1657C	V3A	nsP2	PBS	B1	7	13.68
G3033A	E462K	nsP2	PBS	C4	7	11.68
C3070T	A474V	nsP2	PBS	D3	8	84.68
C3458T	N603N	nsP2	PBS	C1	8	17.81
G3831A	A728T	nsP2	PBS	D2	7	65.92
C4024T	A792V	nsP2	PBS	D1	8	23.38
A4894G	Q288R	nsP3	PBS	B2	7	52.4
C5606G	S525R	nsP3	PBS	D1	8	13.13
G7282A	R527K	nsP4	PBS	C2	7	13.14
G8042-	NA	C	PBS	C4	7	12.52
A8108-	NA	C	PBS	D1	8	15.42
C9481T	Y306Y	E2	PBS	C1	8	16.78
C9850T	S6S	6K	ML336	C4	14	84.78
A9862G	L10L	6K	PBS	C2	7	10.74
			ML336	C4	14	11.56
C10861T	F287F	E1	PBS	A2	4	81.27
			PBS	D3	8	9.52

a NA, not applicable.

### Network analysis of TC-83 in different microenvironments reveals different trajectories of resistance to ML336.

**FIG 7:**
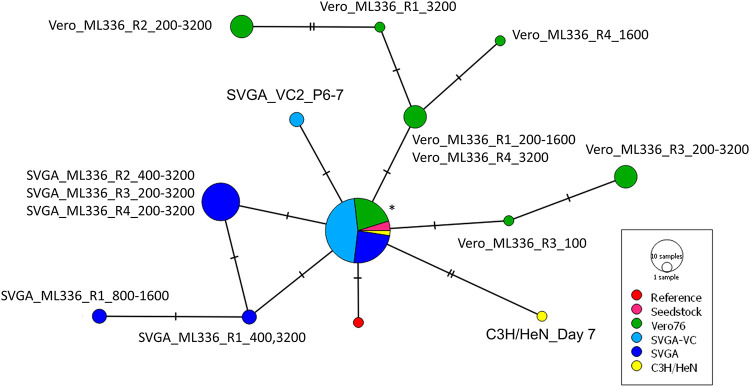
Whole-genome consensus sequence network shows that VEEV TC-83 evolves resistance through different trajectories in distinct microenvironments. Consensus sequences were obtained from NGS sequencing data analysis and then aligned using MUSCLE and plotted based on sequence differences. Samples were grouped and labeled based on virus source. Each tick represents the number of nucleotide differences between sequences. The reference used was taken from GenBank (accession number L10443.1). Samples in the Seedstock, SVGA-VC, and C3H/HeN groups were not treated with ML336. *In vitro* samples are named by cell type, compound or control, and ML336 concentration or passage number. *, Samples include all seed stocks, C3H/HeN day 8, ML336 R1 50-100, ML336 R2 50, ML336 R3 50, ML336 R4 50-800, SVGA R1 50-200, SVGA R2 50-200, SVGA R3 50-100, SVGA R4 50-100, SVGA VC1 P1-7, SVGA VC2 P1-5, and SVGA VC3 1-7.

After determining the major SNPs that emerged in the previous *in vitro* and *in vivo* experiments, we aimed to analyze these data using network analysis. Consensus sequences for the full-length genome for each of the samples from each of the experiments described above, along with the original reference sequence (accession number L01443.1) and sequences from the seed stocks used for passaging, were aligned with the MUSCLE algorithm and plotted using PopART v.1.7, which allowed us to visualize sequence differences (25). We used a minimum spanning tree to plot the sequences, which are shown in Fig. 7. The resulting graph shows that TC-83 lineages diverged based on the host (Vero 76, SVGA, or mouse brain) from which they were isolated. We observed no overlap in trajectory between virus sequences from Vero 76-derived and SVGA-derived samples. In addition, samples in which TC-83 was passaged through SVGA or used to infect C3H/HeN mice, but without addition of ML336, were included, and these samples also resulted in distinct network branches. The network analysis shows the random and ML336-influenced genetic diversity of the VEEV TC-83 virus population in each environment. Of note, the SNPs detected from the wild-type virus diverged from the original consensus in one SVGA-passaged virus and in TC-83 isolated from mouse brains.

## DISCUSSION

In this study, we examined the dynamics of the evolution of resistance to ML336 in VEEV strain TC-83 in three distinct environments. Our experiments were designed to address how different environments for the virus would affect these trajectories. Our network analysis shows that the microenvironment does indeed have an impact on the evolution of drug resistance. We observed similarities between the ML336 resistance-conferring SNPs that emerged in each tested microenvironment and in the timing of emergence; however, the extent of virus genomes containing these SNPs as well as the timing varied between these groups. Moreover, the sites of random SNPs were distinct for each population. As far as we are aware, using network analysis to compare and map the trajectory of viral drug resistance from whole-genome sequencing has not been previously reported.

In previous studies, three mutations that conferred resistance to ML336 were discovered, namely, the Y102C and D116N mutations in the nsP2 gene and the Q210K mutation in the nsP4 gene, with the Q210K mutation causing the strongest loss of potency (5, 6). Because of those prior results, we had expected to find these mutations emerging in the TC-83 population when challenged with ML336 in Vero 76 cells. Notably, while the Q210K mutation was detected in R3, distinct mutations at that amino acid position were found in the other replicates, namely, Q210R and Q210H. In R1, multiple Q210 mutations appeared simultaneously. The changes at Q210 to basic amino acids suggests that the mutation from glutamine to a positively charged amino acid, whether it is lysine, histidine, or arginine, is particularly important in conferring resistance to ML336. The previously discovered Y102C and D116N nsP2 mutations were not found at any passage in any sample when passaging through Vero 76 cells, but when passaging through SVGA cells, the D116N mutation did appear for four passages in one replicate, starting at 100 nM, before disappearing from the population entirely. Interestingly, a mutation at E118V, also in nsP2, appeared in both experiments and followed a similar trajectory of peaking at 100 nM and eventually disappearing. The relative lack of prominent nsP2 SNPs past the first two passages may suggest that D116N and E118V are transitionary mutations that confer resistance but come with a higher fitness cost than the mutations at Q210 in nsP4.

When comparing the SNPs found between the Vero 76 and SVGA passaging experiments, we found that there was not a large difference in terms of the SNPs that arose to significant percentages, as mutations at A461 in nsP3, and Y87, Q210, and R305 appeared frequently throughout all samples, and few new SNPs in the nonstructural genes were found in the SVGA-passaged viruses. However, SNPs from SVGA-passaged TC-83 emerged more slowly than those from Vero-passaged TC-83; with the exception of Q210, most SNPs took three or four passages to reach a consensus in Vero 76 cells, while in SVGA, very few SNPs were able to reach a consensus, even Y87F and R305R. This result suggests that SVGA may provide a modest cellular immune response challenge to TC-83, but it is possible that in both systems the virus overwhelms the cells once the SNPs conferring ML336 resistance emerge and are selected. Remarkably, most of these mutations were not lost in the absence of ML336, which suggests that the fitness cost is not very high, at least for the Q210 mutations where they stayed at near-consensus level regardless of the identity of the other SNPs present. It was difficult to assess the SNPs in C3H/HeN mouse brains due to the low depth of coverage, particularly in genes where SNPs known to confer resistance to ML336 are found. However, the variety of SNPs that were detected between individual untreated mice underscores the genetic diversity of each viral population that is generated during infections. In addition, there was little to no overlap between SNPs found *in vitro* and *in vivo*, again highlighting the differences in microenvironments.

For most of the passaging experiments, we did not confirm whether each major mutation occurred on the same individual viral genomes. However, for each replicate of the Vero 76-ML336 passaging experiment (Fig. 3), we isolated RNA from the final passage, converted it to cDNA, and then ran a PCR using primers that covered the portion of the genome containing all of the major nsP3 and nsP4 mutations, ligated the products into a plasmid system, and ran plasmids through Sanger sequencing. For the most part, each individual sequence contained all of the SNPs that were located in the particular replicate tested (data not shown). This result seems to suggest that, while Q210 mutations may be necessary for resistance to ML336, other SNPs such as Y87F, are often found together on the same viral genomes and may play a role in sustaining viral fitness. Further experiments such as using mutagenesis to create mutant TC-83 clones containing these SNPs or sequencing samples using an Oxford Nanopore MinION sequencer would better determine the relationships between each individual SNP.

The passaging and sequencing methods used in this study are the same as those mentioned in our study on BDGR-4, where Vero 76 cells were used as the passaging medium. In that study, several major SNPs in nsP3 (A461A) and nsP4 (Y87F, Q210K/R/H, E213D, R305R) emerged in those VEEV populations (7). It is not surprising that similar SNPs emerged in this study, as BDGR-4 is a derivative of ML336 within the same amidine structural class. The results reflect that structurally similar compounds elicit similar adaptations in the VEEV genome, although SNPs conferring resistance emerged more slowly to BDGR-4 than they did for ML336. In addition, the data presented here align with data in our previous studies that suggest that ML336 and its analogs directly target the polymerase and disrupt interactions between nsP4 and nsP2 that, in turn, inhibit the synthesis of the positive and negative strands and the subgenomic RNA (5–8). However, it is still unclear what exact effect these mutations have on nsP4, as they all fall under a region where there is no known activity or function throughout all alphaviruses (26).

Since the discovery of ML336 and BDGR-4, we have continued to work on the development of new compounds. Lead candidates will be assessed for resistance in the same manner. Testing the trajectory of resistance in more complex systems, such as in primary neuronal cells, combination therapy, and multiple-cell systems resembling the central nervous system and blood-brain barrier, or in more mouse models, will allow us to examine the effects of more complex environments on antiviral resistance in VEEV in more detail.

## MATERIALS AND METHODS

### Cells and viral culture.

Vero 76 cells (ATCC CCL-131) and SVGA cells (gift from Kui Li, UTHSC) were maintained at 37°C and 5% CO_2_ in complete medium containing Dulbecco modified Eagle medium (DMEM) with Hi-glucose and l-glutamine supplemented with 10% fetal bovine serum (FBS) and 1% penicillin-streptomycin. VEEV TC-83 (lyophilized vaccine) was obtained as a gift from Connie Schmaljohn (United States Army Medical Research Institute of Infectious Diseases, Maryland). Virus seed stocks were amplified in Vero 76 cells in infection medium comprising minimum essential medium with Earle’s salts (MEM) with 2% FBS and 1% penicillin-streptomycin. All reagents and cell culture reagents were purchased from Thermo Fisher Scientific unless otherwise specified.

### Viral quantification.

We used the median tissue culture infectious dose (TCID_50_) to quantify virus in a 96-well-plate format. Plates were seeded with Vero 76 cells overnight. Eleven 10-fold dilutions of virus samples were made, and 100 μl of each dilution was added to each well in triplicate and incubated at 37°C and 5% CO_2_ for 48 h. Following incubation, plates were read visually for cytopathic effect and scored. To confirm visual results, medium was removed, and 12 nM MTT [3-(4,5-dimethylthiazol-2-yl)-2,5-diphenyltetrazolium bromide] (Acros Organics) was added to each well. Plates were incubated for 4 h, after which 10% sodium dodecyl sulfate in 0.01 HCl solution was added to each well. Plates were read at an absorbance wavelength of 570 nm. Wells were scored as positive or negative, and the TCID_50_ was calculated based on the Reed-Muench method (27).

Plaque assays were also used to determine viral titer. Twelve-well plates were seeded with Vero 76 cells overnight. Ten series of 10-fold dilutions of virus samples were made in infection medium, and 200 μl of each dilution was added to each well in duplicate and incubated at 37°C and 5% CO_2_ for 1 h. Following incubation, overlay medium consisting of a 1:1 mix of 2% carboxymethylcellulose and 2×MEM supplemented with 5% FBS, 1% l-glutamine, and 2% penicillin-streptomycin was added to each well. Plates were incubated at 37°C and 5% CO_2_ for 48 h. Wells were fixed with 10% formalin, stained with crystal violet, and washed with Dulbecco’s phosphate-buffered saline, and plaques were counted.

RT-qPCR was used to determine the amount of total viral RNA in tissue samples. cDNA of each sample was amplified using 2× SYBR green Master Mix (Applied Biosystems) and the primers 5′-AGGAGCGCTGAACACTGATGAAGA-3′ and 5′-AGGCGAATTCATGGAAGGG-AGGAT-3′. Reactions were run on the QuantStudio 6 platform (Applied Biosystems) with an initial denaturation step at 95°C for 10 min and 40 cycles of denaturation at 95°C for 15 sec followed by annealing/extension at 60°C for 1 min. Viral RNA copy number was quantified from threshold cycle (*C_T_*) values using a standard curve generated from a linearized plasmid standard containing a partial 250-bp sequence of the nsP2 gene.

### Assay for selection of resistant mutants.

Prior to infection, cells were seeded onto 6-well plates and grown to 90% confluence. Cells were infected with VEEV TC-83 at an initial MOI of 1 or 0.1 as specified in the text and figure legends. Resistant viruses were selected for by passaging wild-type (WT) VEEV TC-83 in 2-fold increasing concentrations of ML336, starting at 50 nM and increasing to 100, 200, 400, 800, 1,600, and lastly, 3,200 nM, for a total of 7 passages. We used 250 μl of supernatant from each passage to initiate the subsequent blind passage. Four distinct replicates were passaged in each experiment. As a control group, VEEV TC-83 was also passaged without compound in triplicate at the same time and in the same manner as the virus was passaged with compound.

### Next-generation sequencing.

To sequence virus genomes from cell culture, 0.5 to 1 ml of supernatant from each passage was harvested and twice-clarified by centrifugation, and RNA was isolated using TRIzol LS reagent (Invitrogen). cDNA was synthesized from purified RNA using random hexamer primers and Superscript IV. cDNA was amplified through 7 or 30 rounds of PCR, using the Phusion high-fidelity kit and a set of 25 primer pairs (Table 1), using a tiling approach that doubly covered the whole genome, making PCR fragments either 500 bp or 1 kb in length. PCR products were purified using the Wizard SV gel and PCR clean-up system (Promega). Libraries for NGS were prepared using the Nextera XT kit (Illumina) and then sequenced on either the Illumina MiSeq or NextSeq 500. Data were analyzed using CLC Genomics Workbench v.12 (Qiagen). To process the sequencing data, reads less than 50 nucleotides in length and with a Phred score of <20 were removed. The remaining reads were mapped to the seed stock consensus sequence, and variants were identified either with a minimum frequency above 1% in all reads at positions with a minimum coverage of 400 or, if the quality and quantity of reads were sufficient, with a minimum frequency above 0.5% in all reads at positions with minimum coverage of 1,000 (19, 20).

### *In vivo* assessment of VEEV TC83 in the presence and absence of ML336 by NGS.

All mouse studies were reviewed by and approved by the UTHSC IACUC in animal care and use protocol number 17.057. Five- to six-week-old, female, C3H/HeN mice were obtained from Charles River Laboratories and randomly assigned to one of three treatment groups as follows: group 1, uninfected control with vehicle; group 2, TC-83 and vehicle only; group 3, TC-83 and ML336 at dosing concentration of 24 mg/kg/day. Mice were dosed via intraperitoneal (i.p.) injection twice per day with vehicle (25% polyethylene glycol 400, 10% Kolliphor RH40, 65% water) or with vehicle containing 12 mg/kg ML336, starting 2 h before infection for 8 days. Mice were infected intranasally (i.n.) with VEEV TC-83 diluted to a concentration of 1 × 10^7^ PFU/30 μl, 15 μl per naris in PBS. PBS was used in place of virus for the vehicle-only control group. Mice were weighed daily and checked twice daily for mortality and morbidity, and subgroups of the VEEV TC-83 infected mice (four mice in each subgroup) were humanely sacrificed on day 4, 7, or 14 postinfection (dpi) or when they either reached >30% weight loss of the starting body weight, or close to 30%, and a clinical score of 0.8 on a euthanasia score sheet approved by the IACUC. Following euthanasia, the whole brain, including the olfactory bulbs, was removed from each mouse. One half of the brain was homogenized in 1 ml of DPBS using the Omni bead mill. We added an equal volume of TRIzol reagent (Invitrogen) to extract total RNA from brain tissue. Because brain tissue has a high lipid content, the RNA from TRIzol was further purified; 2.5 μl of the total RNA, equivalent to 5 mg of homogenized brain originally extracted in TRIzol reagent, was purified with the MagMAX mirVana using the KingFisher Flex system (Thermo Scientific). RNA concentration was measured using a Qubit fluorometer 4 (Invitrogen) using the Qubit RNA HS assay kit (Invitrogen) and stored at –20°C for RT-qPCR to be carried out the following day. Approximately 20 μg of total RNA was obtained from 5 mg of tissue. RT-qPCR was conducted to quantitate viral RNA in mouse brain specimens. Then, 400 ng RNA per sample was taken for assessment using the Superscript III Platinum one-step qRT-PCR kit (Invitrogen) using a TaqMan probe (5′-6-FAM-ACTGGGCTAGGGCTCACAGGCGAG-[TAMRA]-3′) (Applied Biosystems) and primers to the VEEV TC83 nsp2 gene (forward, 5′-AGGAGCGCTGAACACTGATGAAGA-3′; reverse, 5′-AGGCGAATTCATGGAAGGGAGGAT-3′). The RT-qPCR was analyzed on a Quantstudio 6 Flex system for 40 cycles. Viral copy number in the brain samples was calculated using a standard curve made from 10-fold dilutions of VEEV TC83 RNA from the master seed stock. For NGS, the cDNA, PCR amplification and NGS were performed as described above. In brief, mouse brains were normalized as half a brain per ml of DPBS. We measured the *C_T_* values from the brain for the cDNA amplification step. We also normalized the amplicons with our pooling strategy to obtain even coverage. Lastly, we normalized each sample to the same concentration of amplicon prior to library production.

### Network analysis.

The whole-genome consensus sequences from each sample were obtained using CLC Genomics Workbench v.12 (Qiagen). Sequences were aligned using MUSCLE and mapped by the program PopART v.1.7, utilizing a minimum spanning tree to plot each sequence.

### Data availability.

All the NGS data from the studies here are publicly available under BioProject accession number PRJNA655204.
